# Radiological Phenotypes of Bronchiectasis Based on Airway Generation

**DOI:** 10.3390/biomedicines14020337

**Published:** 2026-01-31

**Authors:** Xueqing Yang, Jianping Song, Hongqing Zhang, Nanchuan Jiang, Dongmei Zhang, Zhuanyun Li, Yamin Fan, Yaya Zhou, Weimin Tian, Jianchu Zhang, Wanli Ma, Xiaorong Wang

**Affiliations:** 1 Department of Respiratory and Critical Care Medicine, Union Hospital, Tongji Medical College, Huazhong University of Science and Technology, Wuhan 430022, China; m202376140@hust.edu.cn (X.Y.); songjianpingxh@163.com (J.S.); flanks12345@163.com (H.Z.); 1965463511@qq.com (D.Z.); lizy199299@163.com (Z.L.); 749833758@qq.com (Y.Z.); m202576248@hust.edu.cn (W.T.); zsn0928@163.com (J.Z.); 2 Department of Radiology, Union Hospital, Tongji Medical College, Huazhong University of Science and Technology, Wuhan 430022, China; jiangnanc2000@163.com; 3 Department of Hematology, Union Hospital, Tongji Medical College, Huazhong University of Science and Technology, Wuhan 430022, China; d202482293@hust.edu.cn

**Keywords:** bronchiectasis, radiological phenotype, airway generation

## Abstract

**Background:** High-resolution computed tomography reveals a marked radiological heterogeneity in bronchiectasis; however, the clinical characteristics have not been clearly elucidated. **Method:** We conducted a prospective observational cohort of 334 bronchiectasis patients at Wuhan Union Hospital. Patients were classified into distal airway (DA) and proximal–intermediate airway (PIA) phenotypes and followed every six months for exacerbations. Clinical, inflammatory, microbial, and metabolic features were compared between groups. **Results:** Among 334 patients, 206 were classified as DA and 128 as PIA. Most allergic bronchopulmonary aspergillosis cases belonged to the PIA group (*p* < 0.001). The DA group showed a lower FEV_1_%pred (*p* = 0.010) and Bhalla scores (*p* < 0.001), higher BSI (*p* = 0.003) and FACED scores (*p* < 0.001), more frequent exacerbations (*p* = 0.002), and a greater prevalence of Pseudomonas aeruginosa (PA) colonization (*p* < 0.001). Radiologically, the DA group exhibited more extensive structural lung damage (all *p* < 0.05). Inflammatory profiling showed higher neutrophil counts (*p* = 0.047) and elevated CRP levels (*p* = 0.006) in DA, whereas the PIA group was characterized by eosinophilic inflammation (*p* = 0.026); no significant differences were observed in inflammatory cytokine levels. Microbial interaction network analysis revealed distinct ecological structures between phenotypes. The PIA group showed strong negative correlations with Streptococcus, Rothia, and other commensal taxa, whereas the DA group exhibited no significant associations between Pseudomonas aeruginosa and other species. Furthermore, metabolomic analyses revealed elevated 4-hydroxynonenal levels in the DA group, which also experienced a higher rate of acute exacerbations during follow-up (*p* = 0.003). **Conclusions:** Distinct radiological phenotypes based on airway generation in bronchiectasis are associated with different clinical severity, inflammatory profiles, and microbiome features which enable personalized bronchiectasis management.

## 1. Introduction

Non-cystic fibrosis bronchiectasis (NCFB) is a clinical syndrome characterized by cough, sputum production, and recurrent exacerbations [1], with a heterogeneity in etiology, clinical manifestations, and inflammatory patterns [2]. High-resolution computed tomography (HRCT) highlights the heterogeneity of bronchiectasis, presenting as cylindrical, cystic, and varicose types [3], which was associated with clinical outcomes. Airway involvement also exhibits diversity, ranging from proximal to distal airways [4], which has been shown to represent distinct pathological changes. Distal bronchioles lack marked dilatation but frequently show mucus plug formation and obstruction, characterized by selective MUC5B expression and IL-1β-driven inflammation [5]. In contrast, proximal airways often typically exhibit pronounced ectasia and mucus retention, accompanied by upregulated MUC5B and MUC5AC expressions and prominent neutrophilic inflammation [5]. Furthermore, radiological characteristics can give important clues for etiology [6]. Allergic bronchopulmonary aspergillosis (ABPA) typically presents with central bronchiectasis [7], whereas diffuse panbronchiolitis (DPB) is typically defined by peripheral airway dilation [8]. Thus, the relationship between radiological phenotypes and clinical outcomes in bronchiectasis, beyond the extent of involvement alone, may also reflect different pathological phenotypes that can guide personalized treatment strategies.

Therefore, to further explore the characteristics of patients with distal airway and proximal–intermediate airway involvement, we propose a novel radiological classification of bronchiectasis into distal airway (DA) and proximal–intermediate airway (PIA) groups, based on the generation of bronchial involvement observed in HRCT. We further conducted a comprehensive analysis of the differences between these radiological phenotypes in terms of clinical, microbiological, and metabolomic characteristics, aiming to provide new insights for precise disease assessment and individualized therapeutic strategies in bronchiectasis.

## 2. Materials and Methods

### 2.1. Subjects and Data Collection

This is a prospective observational cohort study that enrolled patients diagnosed with bronchiectasis at Wuhan Union Hospital from 2022 to 2023. Based on the inclusion and exclusion criteria, we ultimately included 334 cases of valid data. Patients were followed up with via telephone every six months to assess the frequency of acute exacerbations, resulting in 227 cases with valid follow-up data. Data collected included basic information, laboratory test results, pulmonary function test data, imaging findings, Saint George’s Respiratory Questionnaire (SGRQ), Leicester Cough Questionnaire (LCQ), blood samples, and sputum samples. Inclusion criteria: (1) age ≥18 years; (2) specimens (sputum and blood) collected during disease stable phase; (3) bronchiectasis confirmed by HRCT. Exclusion criteria: (1) age <18 years; (2) traction bronchiectasis; (3) history of lung surgery; (4) lack of critical clinical information. The project followed the principles of the Declaration of Helsinki and was approved by the Ethics Committee of the Union Hospital of Tongji Medical College, Huazhong University of Science and Technology, without the need for written informed consent (2022–0455), and was registered with ClinicalTrials.gov (ID: NCT05731427).

Bronchiectasis was defined using radiological criteria as follows: an inner or outer airway–artery diameter ratio (the ratio of airway diameter to its adjacent artery diameter) of 1.0 or more, a lack of tapering of the airways, and visibility of airways in the periphery [1]. An acute exacerbation of bronchiectasis was defined as a deterioration in three or more of the following key symptoms for at least 48 h: cough; sputum volume and/or consistency; sputum purulence; breathlessness and/or exercise tolerance; fatigue and/or malaise; and haemoptysis. A clinician determines that a change in bronchiectasis treatment is required [9]. The diagnosis of chronic Pseudomonas aeruginosa infection and severity assessment all follow international expert consensus guidelines [10,11,12,13].

### 2.2. Radiological Phenotypes

**Figure 1:**
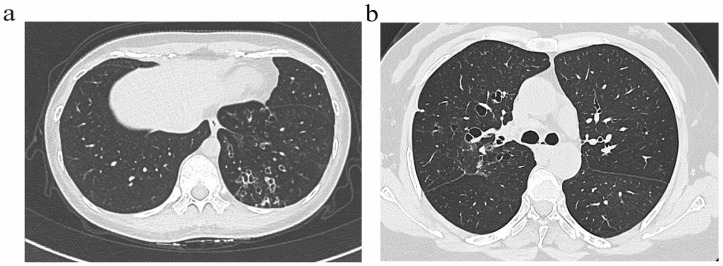
Representative HRCT images illustrating distinct airway phenotype. (**a**) DA phenotype. (**b**) PIA phenotype.

Bronchiectasis assessment followed BTS guidelines [14], excluding traction bronchiectasis. Imaging scoring referenced the Bhalla score [15]. Based on the generation of bronchial involvement demonstrated through HRCT, patients were classified into two radiological phenotypes: (1) The distal airway (DA) group (Figure 1a), defined by dilatation of the distal airway (the sixth generation and beyond). (2) The proximal–intermediate airway (PIA) group (Figure 1b), defined by dilatation of the proximal to intermediate airway (first to fifth generations). If a patient exhibited features of both proximal–intermediate and distal airway phenotypes, classification is determined based on the primary location, extent, and lobar distribution of structural abnormalities on HRCT. In this study, the (25-Bhalla score) was used instead of the total score of lung CT manifestations. For ease of writing, all references to the (25-Bhalla score) below are replaced by the Bhalla score. All images were evaluated by two respiratory physicians and one radiologist independently, who were blinded to the clinical information of patients. Discrepancies were resolved through consensus.

### 2.3. Sputum and Blood Collection and Processing

Sputum samples were collected under clinical supervision. When induction was necessary, nebulized 3% saline was used. Sputum plugs were separated, and samples meeting the criteria of <10 squamous epithelial cells and >25 leukocytes per low-power field were considered valid. Samples were allocated for 16S rRNA sequencing, metabolomics, and inflammatory cytokine assays. Peripheral blood samples were collected via venipuncture in a fasting state. Samples were processed within 2 h of collection. Whole blood was centrifuged at 1500 g for 10 min at 4 °C to separate plasma and serum components. The fractions were aliquoted and stored at −80 °C for subsequent analysis. Plasma samples are primarily used for analyzing oxidative stress markers.

### 2.4. Inflammatory Factor Detection

Inflammatory factors included neutrophil elastase (NE), interleukin-1β (IL-1β), interleukin-6 (IL-6), interleukin-8 (IL-8), interleukin-10 (IL-10), interleukin-17 (IL-17), and tumor necrosis factor-α (TNF-α). All markers were measured according to the operating instructions of the ELISA kits (Elise, [Dmit Bio-Tech Co., Ltd.], [Wuhan, China]). Samples were processed and stored as specified prior to testing. All experimental procedures were performed by trained personnel to ensure the reliability and reproducibility of the results.

### 2.5. 16S rRNA Sequencing and Metabolome Detection

16S rRNA sequencing and untargeted metabolomic profiling were conducted following standard protocols, including DNA extraction, library preparation, and LC–MS-based metabolite detection (see Supplementary Materials for details).

### 2.6. Data Analysis

All data obtained during the study underwent preprocessing. Missing data were imputed using multiple imputation methods. Continuous variables that met the assumption of normal distribution were analyzed using Student’s *t*-test, with results expressed as mean ± standard deviation (mean ± SD). Continuous variables that did not meet the assumption of normal distribution were analyzed using the Mann–Whitney U test, with results expressed as interquartile range (IQR). Categorical variables were analyzed using chi-square tests, with results expressed as frequencies (%). Statistical analysis of data was performed using SPSS 27.0 (SPSS Inc., Chicago, IL, USA), GraphPad Prism 9.0 (GraphPad Software Inc., San Diego, CA, USA), and R for Windows 4.4.1 (https://r-project.org; accessed on 10 October 2025). All statistical analyses were two-tailed, with statistical significance set at *p* < 0.05.

## 3. Results

### 3.1. Demographics and Clinical Characteristics of the Patients

This study included 334 patients diagnosed with bronchiectasis who were treated at Wuhan Union Hospital. Among these, 206 cases (61.7%) belonged to the DA group, while 128 cases (38.3%) belonged to the PIA group. No statistically significant differences existed between the groups in terms of gender, age, body mass index (BMI), or comorbidities, although smoking was more prevalent in the DA group. In terms of etiology, idiopathic bronchiectasis was the most frequent cause in both groups. Importantly, 95% of ABPA patients were classified as the PIA group (*p* < 0.001). No differences were observed in other etiological phenotypes. Basic information on bronchiectasis patients is presented in Table 1.

### 3.2. Association Between Radiological Phenotypes and Clinical Outcomes

**Figure 2:**
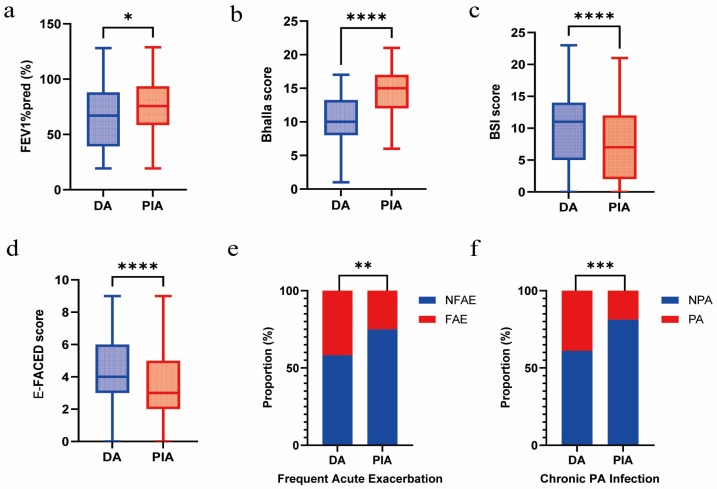
Association between radiological phenotypes and clinical outcomes. (**a**) Comparison of FEV1%pred between DA and PIA phenotypes. (**b**) Comparison of Bhalla score between DA and PIA phenotypes. (**c**) Comparison of BSI score between DA and PIA phenotypes. (**d**) Comparison of E-FACED score between DA and PIA phenotypes. (**e**) Comparison of frequent acute exacerbation between DA and PIA phenotypes. (**f**) Comparison of chronic PA infection between DA and PIA phenotypes. (* *p* < 0.05; ** *p* < 0.01; *** *p* < 0.001; **** *p* < 0.0001).

Compared with patients in the PIA group, those in the DA group exhibited a lower FEV1%pred (*p* = 0.010) and Bhalla score (*p* < 0.001) and a higher BSI score (*p* = 0.003) and E-FACED score (*p* < 0.001). In addition, they showed more frequent acute exacerbations (*p* = 0.002) and a significantly higher prevalence of chronic PA infection (*p* < 0.001) (Figure 2a–f).

Radiologically, compared with the PIA group, the DA group exhibited more extensive structural lung damage (Supplementary Table S1), characterized by higher proportions of cystic and mixed bronchiectasis (*p* = 0.035), more pronounced bronchial dilatation (*p* < 0.001), a greater extent of bronchiectatic involvement (*p* < 0.001), and a more marked bronchial wall thickening (*p* < 0.001). Concurrently, the DA group exhibited more pronounced small airway-related features: higher mucus plugs (*p* < 0.001), an increased prevalence of bullae (*p* = 0.030), a greater occurrence of mosaic sign (*p* = 0.031), and more frequent collapse or consolidation (*p* < 0.001). However, no significant difference in the presence of tree-in-bud signs was observed between the two groups (*p* = 0.264).

### 3.3. Association Between Radiological Phenotypes, Mucus Burden, and Inflammation

**Figure 3:**
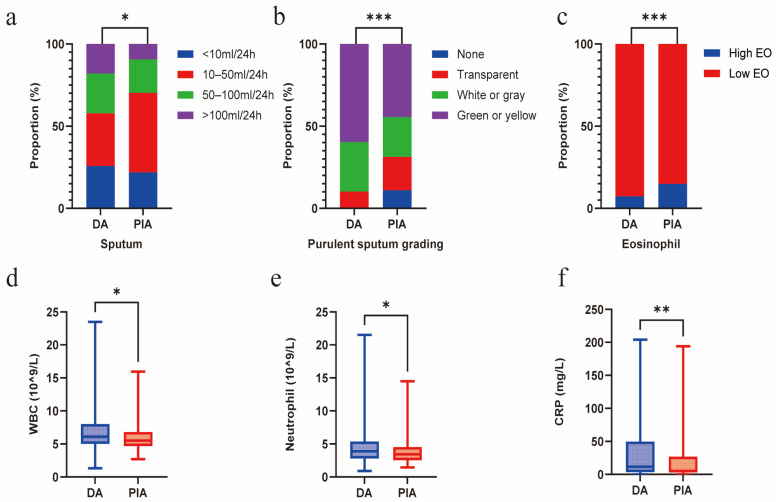
Radiological phenotypes correlate with mucus burden and inflammation. (**a**) Comparison of sputum volume between DA and PIA phenotypes. (**b**) Comparison of purulent sputum grading between DA and PIA phenotypes. (**c**) Comparison of eosinophil levels between DA and PIA phenotypes. (**d**) Comparison of WBC count between DA and PIA phenotypes. (**e**) Comparison of neutrophil count between DA and PIA phenotypes. (**f**) Comparison of CRP levels between DA and PIA phenotypes. (* *p* < 0.05; ** *p* < 0.01; *** *p* < 0.001).

Compared with the PIA group, patients in the DA group exhibited a significantly greater mucus burden, characterized by a higher sputum volume (*p* = 0.015) and a higher grade of purulent sputum (*p* < 0.001). Concurrently, a significantly higher proportion of the PIA group exhibited blood eosinophil counts exceeding 300 cells/μL (*p* = 0.026). In contrast, the DA group exhibited higher systemic inflammation levels, manifested as elevated white blood cell (WBC) counts (*p* = 0.017) and C-reactive protein (CRP) levels (*p* = 0.035), primarily due to the increased neutrophil count (*p* = 0.047) (Figure 3a–f). No significant differences in sputum inflammatory cytokine levels were observed between the two groups (Supplementary Table S2).

### 3.4. Association Between Radiological Phenotypes and Microbiota

**Figure 4:**
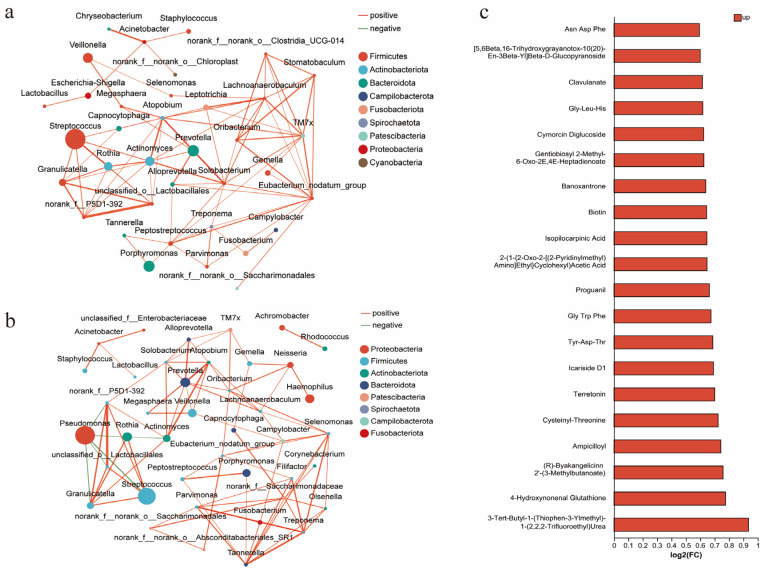
Radiological phenotypes correlate with microbiota and metabolites. (**a**) Interrelationships of airway microbiota in the DA phenotype. (**b**) Interrelationships of airway microbiota in the PIA phenotype. (**c**) Association between radiological phenotypes and metabolites. The bar plot shows metabolites upregulated in the DA group compared with the PIA group. FC (fold change) represents the ratio of metabolite abundance in the DA group relative to the PIA group, presented as log_2_-transformed fold changes [log2(FC)]. Up denotes metabolites that are increased in the DA group.

In this study, 178 quality-assured sputum samples were collected and analyzed via 16S rRNA sequencing. The results showed no significant differences in α-diversity metrics between groups (Supplementary Table S3), but distinct differences in dominant microbial communities. Streptococcus (*p* < 0.001) and Prevotella (*p* = 0.007) abundances were significantly higher in the PIA group than in the DA group, while the PA abundance was markedly elevated in the DA group (*p* = 0.008). A further analysis of the microbial interaction network analysis further highlighted distinct ecological structures. The DA group demonstrated a predominantly positively correlated microbial network, while PA showed no significant associations with other species (Figure 4a). In contrast, in the PIA group, PA exhibited strong negative correlations with Streptococcus, Rothia, and other commensal taxa (Figure 4b).

### 3.5. Association Between Radiological Phenotypes and Metabolism

Metabolomic analysis revealed significantly elevated levels of multiple short peptides in the DA group compared to the PIA group (Figure 4c). Notably, 4-Hydroxynonenal (4-HNE) was markedly increased in the DA group. In plasma, the DA group also showed higher glutathione (GSH, *p* = 0.003) and catalase levels (CAT, *p* = 0.004), whereas other oxidative stress markers, including malondialdehyde (MDA), hydrogen peroxide (H_2_O_2_), and other plasma or sputum indicators, did not show significant differences (Supplementary Table S4).

### 3.6. Association Between Radiological Phenotypes and Follow-Up Acute Exacerbation

**Figure 5:**
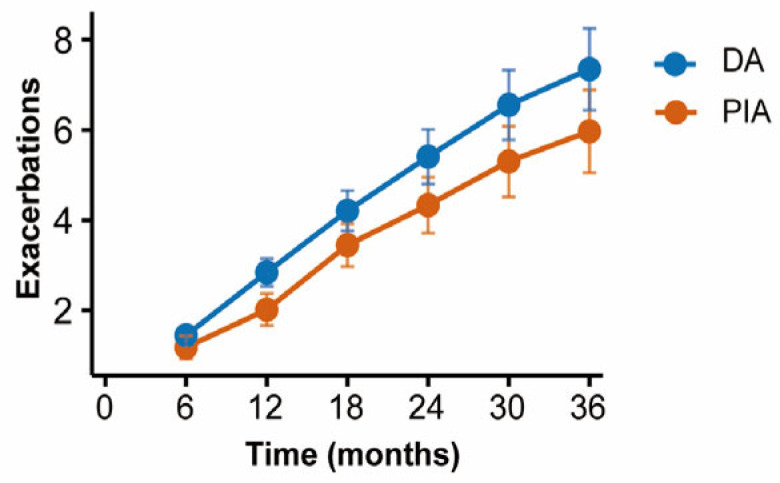
Comparison of cumulative exacerbations between radiological phenotypes during follow-up.

Patients were followed up with every six months to record the frequency of acute exacerbations. Cumulative incidence curves demonstrated that patients in the DA group experienced significantly more frequent exacerbations compared with those in the PIA group (*p* = 0.003) (Figure 5).

## 4. Discussion

Based on 334 patients with NCFB, our study is the first to propose a classification of PIA and DA phenotypes according to the airway generation involvement observed through HRCT. We found that patients with peripheral airway involvement exhibited higher systemic inflammation, more mucus burden, and poorer clinical outcomes. This classification not only highlights the radiological heterogeneity of bronchiectasis, but also emphasizes the important role of the peripheral airways in disease progression, providing evidence for identifying high-risk patients and justifying targeted clinical management strategies.

Airway generation has gradually emerged as a key focus in the diagnosis and classification of respiratory diseases. As early as 1995, Reiff et al. categorized bronchiectasis imaging into central, peripheral, or mixed types based on the midpoint between the hilum and chest wall, and found a significantly increased proportion of central bronchiectasis in ABPA [16]. Kim S et al. further classified severe asthma into large airway and small airway types using subsegmental bronchi as the boundary, and found that SA patients had the most severe airflow limitation and required higher maintenance treatment [17]. Furthermore, small airways have increasingly attracted attention for their pivotal role in the pathogenesis and progression of NCFB. Their dysfunction often arises before the emergence of noticeable symptoms, spirometric abnormalities and imaging findings hence termed the “silent zone” [18]. However, small airways measuring less than 1–2 mm cannot be accurately identified through conventional HRCT, while the sixth-level airways represent the outermost level that can be relatively stably and clearly visualized in routine HRCT [19]. This level is also emphasized in the Bhalla score15. Accordingly, patients with bronchiectasis involving the sixth-generation bronchi and beyond were defined as having a DA group, with small airway involvement incorporated into this phenotype. Interestingly, our study found that this phenotype was strongly associated with major clinical outcomes, highlighting its potential as a distinct and independent clinical subtype.

The distribution of bronchiectasis also offer clues into the potential etiology [20]. Our study found that 95% of ABPA patients were classified into the PIA group, and central bronchiectasis has been recognized as one of the key diagnostic criteria for ABPA [21]. In contrast, DPB primarily affects the peripheral airways, presenting with tree-in-bud lesions, lobular nodules, and basal or diffuse distributions, whereas Primary Ciliary Dyskinesia (PCD) patients frequently exhibit marked atelectasis, mucus plugging, and budding changes [22]. Due to sample limitations, our study did not include DPB and PCD patients; further research is needed for validation.

Mucus is not only a significant predictor of acute exacerbations in bronchiectasis but is also closely linked to an increased mortality risk [23]. In our study, patients in the DA group exhibited a more extensive mucus burden, including an increased sputum volume, higher purulent sputum grading, and more extensive mucus plug formation. Although no significant differences in the “tree-in-bud sign” were observed across groups, the DA group still exhibited a heavier overall burden. This distinctive pattern can be attributed to the anatomical and physiological differences between proximal and peripheral airways. Under pathological conditions, an increased MUC5B expression in the peripheral airways [5], coupled with shortened and fewer cilia, synergistically contributes to mucus clearance impairment and accumulation [24,25], highlighting the need for targeted therapies to enhance mucus clearance [26]. In contrast, lesions in the PIA group are primarily confined to the proximal airways, which enable an efficient mucus clearance through the combined action of cilia and the cough reflex [27]. Mucus in the proximal airways can be effectively cleared through traditional methods including postural drainage, percussion therapy, and high-frequency chest wall oscillation [28]. These findings highlight the differences in mucus burden and therapeutic strategies between the two radiological phenotypes [29]. Notably, chronic PA colonization was significantly more common in the DA group, likely reflecting impaired mucociliary clearance and hypoxic microenvironments in distal airways [30]. Consistent with this observation, 16S rRNA sequencing further confirmed the dominant role of PA in the DA phenotype. Further microbial interaction network analysis showed a markedly weakened competitive inhibition surrounding PA in the DA group. This pattern aligns with the findings reported by Micheál Mac Aogáin et al., where frequent exacerbators exhibited reduced negative correlations between PA and commensal taxa, indicating a disruption of microbial network stability [31].

The inflammatory response has been recognized as a core mechanism in the development and progression of bronchiectasis, with small airways potentially serving as the initial site of lung injury caused by irritant or inflammatory inhalants [32]. Our study also confirms that patients in the DA group exhibit elevated systemic inflammatory markers, including increased neutrophil counts and CRP levels. Therapies targeting neutrophilic inflammation such as DPP-1 inhibitors may offer greater clinical benefits [33]. In contrast, in the PIA group, a greater proportion of patients had blood eosinophil counts over 300 cells/μL, indicating more pronounced eosinophilic inflammation [34]. Its treatment may benefit from interventions targeting the Th2 pathway, such as inhaled corticosteroids (ICS) or anti-Th2 monoclonal antibodies [35]. However, the PIA group included some ABPA patients with known eosinophilic inflammation [21], which may introduce selection bias, requiring further validation. Nevertheless, these findings still highlight the association between radiological phenotypes and inflammatory endotypes, indicating that personalized anti-inflammatory therapy based on these phenotypes holds potential application value in patients with bronchiectasis [36].

However, our study also has limitations. First, our research sample was derived from a single center, potentially introducing selection bias. Second, while we classified radiological phenotypes through the generation of bronchial involvement alone, this approach fails to adequately reflect the functional state of small airways and struggles to demonstrate airway continuity. Finally, the underlying mechanisms driving the differences between the radiological phenotypes were not fully explored in our study. Future research should employ longitudinal, multicenter cohort designs and integrate functional assessment tools to overcome these limitations.

## 5. Conclusions

In summary, our study first proposes DA and PIA phenotypes based on bronchial generation observed through HRCT, demonstrating that distinct radiological phenotypes in bronchiectasis are associated with different clinical severities, inflammatory profiles, and microbiome features. Phenotype-based stratification may guide personalized management and targeted interventions in bronchiectasis.

## Figures and Tables

**Table 1:** Comparison of basic information of bronchiectasis.

Variables	All Patients (*n* = 334)	DA (*n* = 206)	PIA (*n* = 128)	*p* Value
Male/Female, *n*	128/206	72/134	56/72	0.108
Age (median, IQR, years)	58 (49, 67)	58.5 (51, 69)	56 (47.25, 66)	0.061
BMI (median, IQR, kg/m^2^)	20.82 (18.37, 23.75)	20.77 (18.21, 23.63)	21.01 (18.60, 24.19)	0.207
Ever-smoker, *n* (%)	49 (14.7%)	23 (11.2%)	26 (20.3%)	0.022
Disease etiology, *n* (%)				
No_cause_identified	125 (37.4%)	80 (38.8%)	45 (35.2%)	0.576
Post_infectious	87 (26.0%)	60 (29.1%)	27 (21.1%)	0.134
Tuberculosis	30 (9.0%)	19 (9.2%)	11 (8.6%)	1.000
NTM	8 (2.4%)	6 (2.9%)	2 (1.6%)	0.715
ABPA	20 (6.0%)	1 (0.5%)	19 (14.8%)	<0.001
COPD	28 (8.4%)	19 (9.2%)	9 (7.0%)	0.617
Asthma	9 (2.7%)	4 (1.9%)	5 (3.9%)	0.312
Immune_defect	3 (0.9%)	1 (0.5%)	2 (1.6%)	0.561
Other *	24 (7.2%)	16 (7.8%)	8 (6.3%)	0.761
Comorbidity, *n* (%)				
COPD	87 (26.0%)	54 (26.2%)	33 (25.8%)	0.930
Asthma	13 (3.9%)	5 (2.4%)	8 (6.3%)	0.079
Tuberculosis	38 (11.4%)	28 (13.6%)	10 (7.8%)	0.106
Sinusitis	27 (8.1%)	19 (9.2%)	8 (6.3%)	0.332
Treatment, *n* (%)				
Long-term antibiotic therapy	31(9.3%)	19 (9.2%)	12 (9.4%)	0.963
Glucocorticoid use	25 (7.5%)	19 (9.2%)	6 (4.7%)	0.126
Bronchodilator use	58 (17.4%)	41 (19.9%)	17 (13.3%)	0.120

Definition of abbreviations: BMI = body mass index; NTM = nontuberculous mycobacteria; ABPA = allergic bronchopulmonary aspergillosis; COPD = chronic obstructive pulmonary disease. Data presented as media (IQR) where applicable. * Other diseases include inflammatory bowel disease, rheumatoid arthritis, Sjögren’s syndrome, and other autoimmune disorders.

## Data Availability

The dataset used and analyzed in this study is available from the corresponding authors upon reasonable request.

## Supplementary Materials

The following supporting information can be downloaded at https://www.mdpi.com/article/10.3390/biomedicines14020337/s1. Table S1: Differences in HRCT Across Radiological Phenotypes; Table S2: Differences in Inflammatory Cytokine Across Radiological Phenotypes; Table S3: Differences in Microbial Diversity and Community Composition Across Radiological Phenotypes; Table S4: Differences in Oxidative Stress Biomarkers Across Radiological Phenotypes.

## Abbreviations

The following abbreviations are used in this manuscript:

ABPA	Allergic bronchopulmonary aspergillosis
BMI	Body mass index
CRP	C-reactive protein
CAT	Catalase levels
DA	Distal airway
DPB	Diffuse panbronchiolitis
GSH	Glutathione
HRCT	High-resolution computed tomography
H_2_O_2_	Hydrogen peroxide
ICS	Inhaled corticosteroids
IL-1β	Interleukin-1β
IL-6	Interleukin-6
IL-8	Interleukin-8
IL-10	Interleukin-10
IL-17	Interleukin-17
IQR	Interquartile range
LCQ	Leicester Cough Questionnaire
MDA	Malondialdehyde
NCFB	Non-cystic fibrosis bronchiectasis
NE	Neutrophil elastase
PA	Pseudomonas aeruginosa
PCD	Primary Ciliary Dyskinesia
PIA	Proximal–intermediate airway
SGRQ	Saint George’s Respiratory Questionnaire
SD	Standard deviation
TNF-α	Tumor necrosis factor-α
WBC	White blood cel
4-HNE	4-Hydroxynonenal
