# Supplementary material for: Radiological Phenotypes of Bronchiectasis Based on Airway Generation
Source: Biomedicines. 2026 Jan 31;14(2):337. doi: 10.3390/biomedicines14020337 (PMC12938277; doi:10.3390/biomedicines14020337)
Supplement: Supplementary file 1 [file biomedicines-14-00337-s001.zip › biomedicines-4052973-supplementary.pdf]

Supplementary Materials

1. Supplementary methods

1.1 16S rRNA gene sequencing of sputum samples

1.2 Metabolome detection of sputum samples

2. Supplementary tables

Table S1. Differences in HRCT Across Radiological Phenotypes

Table S2. Differences in Inflammatory Cytokine Across Radiological Phenotypes

Table S3. Differences in Microbial Diversity and Community Composition Across Radiological Phenotypes

Table S4. Differences in Oxidative Stress Biomarkers Across Radiological Phenotypes

1. Supplementary methods

1.1 16S rRNA gene sequencing of sputum samples

Microbial community genomic DNA was extracted from sputum using the E.Z.N.A. Soil DNA Kit (Omega Bio-tek, Norcross, GA, USA) according to the manufacturer's instructions. DNA quality and concentration were assessed via 1.0% agarose gel electrophoresis and the NanoDrop2000 spectrophotometer (Thermo Scientific, USA), then stored at -80 ° C for subsequent use. The hypervariable region V3-V4 of the bacterial 16S rRNA gene was amplified using the ABI GeneAmp 9700 system with the primer pair 338F(5'-ACTCCTACGGGAGGCAGCAG-3') and 806R(5'-GGACTACHVGGGTWTCTAAT-3') using an ABI GeneAmp 9700 PCR thermal cycler (ABI, California, USA). PCR amplification conditions were as follows: initial denaturation at 95 ° C for 3 minutes, followed by 27 cycles (denaturation at 95 ° C for 30 seconds, annealing at 55 ° C for 30 seconds, extension at 72 ° C for 45 seconds), concluding with a single extension at 72 ° C for 10 minutes and termination at 4 ° C. PCR products were separated by 2% agarose gel electrophoresis, purified using the AxyPrep DNA Gel Extraction Kit (Axygen Biosciences, Union City, CA, USA) according to manufacturer instructions, and quantified with Qubit 4.0 (Promega, USA).

Purified amplified fragments were mixed at equimolar concentrations and subjected to paired-end sequencing on the Illumina NextSeq PE300 platform/NovaSeq PE250 platform by Majorbio Biotechnology Co., Ltd. (Shanghai, China) following standard procedures. Raw 16S rRNA gene sequencing reads underwent demultiplexing and quality filtering using fastp v0.20.0, followed by assembly with FLASH v1.2.7 under the following criteria: (i) Truncation of 300 bp reads: Truncate reads when the average quality score within a 50 bp sliding window is <20. Reads shorter than 50 bp after truncation and reads containing ambiguous characters were discarded. (ii) Assembly performed only on reads with overlapping sequences >10 bp, which were joined based on their overlapping regions. The maximum mismatch rate in overlapping regions was set to 0.2. Unassemblable reads were discarded; (iii) Samples were distinguished based on barcodes and primers, with sequence orientation adjusted. Barcode sequences required exact matches, while primer sequences allowed two nucleotide mismatches. UPARSE version 7.1 was used to cluster operational taxonomic units (OTUs) at a 97% similarity threshold, identifying and removing chimeric sequences. The RDP Classifier version 2.2 was employed to align sequences against the 16S rRNA database (confidence threshold 0.7) and analyze the taxonomic characteristics of representative sequences for each OTU.

1.2 Metabolome detection of sputum samples

1.2.1 Metabolite Extraction

Pipette 100 μ L of liquid sample into a 1.5 mL centrifuge tube. Add 400 μ L of extraction solvent (acetonitrile:methanol = 1:1) containing four internal standards (L-2-chlorophenylalanine (0.02 mg/mL), etc.). Vortex for 30 seconds, then perform low-temperature ultrasonic extraction for 30 minutes (5 ° C, 40 KHz). Allow the sample to stand at -20 ° C for 30 minutes. Centrifuge at 4 ° C, 13,000 g for 15 min. Transfer the supernatant, evaporate to dryness under nitrogen, redissolve in 100 μL of redissolution solvent (acetonitrile:water = 1:1), perform low-temperature ultrasonic extraction for 5 minutes (5 ° C, 40 kHz), centrifuge at 4 ° C, 13,000 g for 10 minutes. The supernatant was transferred to sample vials for LC-MS/MS analysis.

1.2.2 Quality control sample

Equal volumes of all sample metabolites were pooled to prepare quality control (QC) samples. During instrumental analysis, one QC sample was inserted every 5 – 10 samples to assess the repeatability of the entire analytical process.

1.2.3 UPLC-MS/MS analysis

The LC-MS/MS analysis of sample was conducted on a SCIEX UPLC-Triple TOF 5600 system equipped with an ACQUITY HSS T3 column (100 mm × 2.1 mm i.d., 1.8 μm; Waters, USA) at Majorbio Bio-Pharm Technology Co. Ltd. (Shanghai, China). 3 μL sample was separated on an HSS T3 column (100 mm × 2.1 mm i.d., 1.8 μm) before entering mass spectrometry detection. Mobile phase A consists of a water/acetonitrile (95/5, v/v) solution containing 0.1% formic acid. Mobile phase B is an acetonitrile/isopropanol/water (47.5/47.5/5, v/v/v) solution containing 0.1% formic acid. MS conditions: Sample mass spectrometry signal acquisition employs positive and negative ion scanning modes, with a mass scanning range of m/z: 50-1000. The positive mode ion spray voltage is 5000 V, the negative mode ion spray voltage is -4000 V, the declustering voltage is 80 V, the spray gas pressure is 50 psi, the auxiliary heating gas pressure is 50 psi, the curtain gas pressure is 30 psi, the ion source heating temperature is 550° C, and the collision energy is cycled between 20-60 V. Heater gas 50 psi, curtain gas 30 psi, ion source heating temperature 550° C, 20-60 V cyclic collision energy.

1.2.4 Data analysis

After instrument completion, raw LC-MS data were imported into the metabolomics processing software Progenesis QI (Waters Corporation, Milford, USA) for baseline filtering, peak identification, integration, retention time correction, and peak alignment. This ultimately yielded a data matrix comprising retention time, mass-to-charge ratio, and peak intensity. Concurrently, MS and MS/MS mass spectrometry information was matched against public metabolite databases HMDB (http://www.hmdb.ca/) and Metlin (https://metlin.scripps.edu/), as well as Meiji's proprietary database, to obtain metabolite identifications.

Upload the searched data matrix to the MajorBio Cloud platform (cloud.majorbio.com) for analysis. First, preprocess the data matrix as follows: Remove missing values using the 80% rule, retaining variables with non-zero values in at least 80% of samples. Then impute missing values (using the minimum value in the original matrix to fill gaps). To minimize errors from sample preparation and instrument instability, normalize the response intensity of sample mass spectrometry peaks using the sum normalization method, yielding a normalized data matrix. Simultaneously, variables with QC sample relative standard deviation (RSD) >30% are removed, and log10 transformation is applied to obtain the final data matrix for subsequent analysis. Subsequently, the ropls package (Version 1.6.2) in R was employed to perform principal component analysis (PCA) and orthogonal partial least squares discriminant analysis (OPLS-DA) on the preprocessed data matrix. Model stability was assessed using 7-fold cross-validation. Significantly different metabolites were selected based on variable importance in projection (VIP) values from the OPLS-DA model and Student's t-test p-values. Metabolites with VIP > 1 and $p < 0.05$ were identified as significantly different.

Differentially expressed metabolites underwent metabolic pathway annotation via the KEGG database (https://www.kegg.jp/kegg/pathway.html) to identify pathways involving these metabolites. Pathway enrichment analysis was performed using the Python package scipy.stats, with the biological pathways most relevant to the experimental treatments identified through Fisher's exact test.

Table S1. Differences in HRCT Across Radiological Phenotypes

Variables	All patients (*n* =334)	DA (*n* =206)	PIA (*n* =128)	*p* value
Types of bronchiectasis, *n* (%)				0.035

Columnar bronchiectasis	50（15.0%）	23（11.2%）	27（21.1%）	
Varicose/Cystic bronchiectasis	110（32.9%）	74（35.9%）	36（28.1%）	
Both	174（52.1%）	109（52.9%）	65（50.8%）	
Degree of bronchiectasis（bronchial to accompanying pulmonary arterial diameter ratio), *n* (%)				＜ 0.001
＞1	77（23.1%）	25（12.1%）	52（40.6%）	
2-3	153（45.8%）	96（46.6%）	57（44.5%）	
＞3	104（31.1%）	85（41.3%）	19（14.8%）	
Extension of bronchiectasis (number of affected segments), *n* (%)				＜ 0.001
1-5	141（42.2%）	66（32.0%）	75（58.6%）	
6-9	112（33.5%）	75（36.4%）	37（28.9%）	
>9	81（24.3%）	65（31.6%）	16（12.5%）	
Thickness of bronchial wall (bronchial wall to concomitant arterial thickness ratio), *n* (%)				＜ 0.001
Normal range	21（6.3%）	8（3.9%）	13（10.2%）	
1	102（30.5%）	50（24.3%）	52（40.6%）	
1-2	136（40.7%）	85（41.3%）	51（39.8%）	
＞2	75（22.5%）	63（30.6%）	12（9.4%）	
Number of lung segments with mucus plugs, *n* (%)				＜ 0.001
0	79（23.7%）	33（16.0%）	46（35.9%）	
1-5	116（34.7%）	70（34.0%）	46（35.9%）	
6-9	94（28.1%）	65（31.6%）	29（22.7%）	
＞9	45（13.5%）	38（18.4%）	7（5.5%）	
Number of lung segments with Tree-in-bud sign, *n* (%)				0.264
0	109（32.6%）	68（33.0%）	41（32.0%）	
1-5	91（27.2%）	55（26.7%）	36（28.1%）	
6-9	80（24.0%）	55（26.7%）	25（19.5%）	
＞9	54（16.2%）	28（13.6%）	26（20.3%）	
Sacculations(number of affected segments), *n* (%)				＜ 0.001
0	112（33.5%）	54（26.2%）	58（45.3%）	
1-5	116（34.7%）	69（33.5%）	47（36.7%）	

6-9	69（20.7%）	51（24.8%）	18（14.1%）	
＞9	37（11.1%）	32（15.5%）	5（3.9%）	
Number of lung bullae, *n* (%)				0.03
0	223（66.8%）	147（71.4%）	76（59.4%）	
＜=4(unilateral)	45（13.5%）	21（10.2%）	24（18.8%）	
＜=4(bilateral)	26（7.8%）	18（8.7%）	8（6.2%）	
＞4(bilateral)	40（12.0%）	20（9.7%）	20（15.6%）	
Number of lung segments with emphysema, *n* (%)				0.654
0	144（43.1%）	85（41.3%）	59（46.1%）	
1-5	102（30.5%）	66（32.0%）	36（28.1%）	
＞5	88（26.3%）	55（26.7%）	33（25.8%）	
Number of lung segments with Mosaic sign, *n* (%)				0.031
0	250（74.9%）	143（69.4%）	107（83.6%）	
1-5	49（14.7%）	38（18.4%）	11（8.6%）	
6-9	16（4.8%）	11（5.3%）	5（3.9%）	
＞9	19（5.7%）	14（6.8%）	5（3.9%）	
Collapse/consolidation, *n* (%)				＜0.001
Absent	127（38.0%）	64（31.1%）	63（49.2%）	
Subsegmental	79（23.7%）	47（22.8%）	32（25.0%）	
Segmental/lobar	128（38.3%）	95（46.1%）	33（25.8%）	
Bhalla score, *n* (%)				
Median (IQR)	12 (9,15)	10 (8, 13.25)	15 (12, 17)	＜0.001
16-25 (*n*, %)	57（17.1%）	14（6.8%）	43（33.6%）	
8-15 (*n*, %)	207（62.0%）	134（65.0%）	73（57.0%）	
＜8 (*n*, %)	70（21.0%）	58（28.2%）	12（9.4%）	

Table S2. Differences in Inflammatory Cytokine Across Radiological Phenotypes

Variables	All patients (*n* =125)	DA (*n* =89)	PIA (*n* =36)	*p* value
Inflammatory cytokines				
NE (median, IQR, ng/mL)	29.00 (21.60, 38.44)	29.00 (22.37, 38.40)	29.54 (21.06, 38.56)	0.873
IL-1β (median, IQR, pg/mL)	1022.59 (382.60, 3641.56)	1024.85 (409.97, 3303.81)	1022.59 (217.78, 3962.03)	0.863
IL-6 (median, IQR, pg/mL)	42.89 (23.54, 92.00)	43.31 (23.58, 92.00)	40.67 (21.64, 91.10)	0.298

IL-8 (median, IQR, pg/mL)	9803.92 (998.67, 16596.00)	8501.39 (976.25, 16047.98)	12282.10 (1069.51, 16596.00)	0.479
IL-10 (median, IQR, pg/mL)	170.80 (168.24, 172.58)	170.80 (168.24, 172.58)	170.69 (167.41, 172.58)	0.456
IL-17 (median, IQR, pg/mL)	326.22 (297.04, 341.25)	327.38 (305.53, 336.30)	323.36 (297.04, 346.31)	0.651
TNF-α (median, IQR, pg/mL)	690.07 (229.00, 798.33)	690.36 (240.78, 785.61)	685.49 (193.64, 1157.82)	0.946

Definition of abbreviations: NE= neutrophil elastase; IL-1 β = interleukin-1 beta; IL-6= interleukin-6; IL-8= interleukin-8; IL-10= interleukin-10; IL-17= interleukin-17; TNF- α = tumor necrosis factor-alpha

Data presented as media (IQR) where applicable.

Table S3. Differences in Microbial Diversity and Community Composition Across Radiological Phenotypes

Variables	All patients (*n* =178)	DA (*n* =118)	PIA (*n* =60)	*p* value
Microbial alpha diversity				
Sobs (median, IQR)	220.00 (165.00, 274.00)	220.00 (163.00, 270.25)	217.00 (169.25, 284.50)	0.444
Shannon (median, IQR)	0.89 (0.13, 2.47)	1.09 (0.16, 2.51)	0.48 (0.13, 2.40)	0.115
Simpson (median, IQR)	0.41 (0.18, 0.75)	0.37 (0.18, 0.75)	0.44 (0.19, 0.75)	0.599
Ace (median, IQR)	305.03 (214.87, 398.16)	323.83 (230.27, 395.99)	290.13 (201.65, 409.03)	0.218
Chao (median, IQR)	303.56 (222.49, 384.65)	306.50 (222.49, 386.14)	299.60 (223.29, 374.00)	0.847
Microbial abundance	n=178	n=118	n=60	
Streptococcus (median, IQR, %)	16.77 (2.88, 35.73)	15.72 (2.96, 35.56)	20.69 (2.72, 37.43)	＜ 0.001
Pseudomonas (median, IQR, %)	4.14 (0.02, 48.61)	9.45 (0.11, 52.35)	0.74 (0.01, 40.31)	0.008
Prevotella (median, IQR, %)	3.90 (1.05, 12.56)	2.90 (0.69, 10.64)	5.42 (1.85, 14.23)	0.007
Haemophilus (median, IQR, %)	0.20 (0.00, 2.98)	0.21 (0.00, 3.54)	0.19 (0.01, 2.05)	0.455

Table S4. Differences in Oxidative Stress Biomarkers Across Radiological Phenotypes

Variables	All patients (n=144)	DA (n=100)	PIA (n=44)	*p* value
Oxidative stress level				
Total Nitrate Concentration (median,	418.92 (48.65, 1085.93)	531.19 (48.65, 914.77)	303.72 (48.65, 1324.32)	0.709

IQR, μmol/L)				
NO_2(median, IQR, μmol/L)	298.31 (79.64, 557.95)	326.63 (134.31, 557.95)	210.36 (39.88, 599.52)	0.171
NO_3(median, IQR, μmol/L)	60.97 (6.42, 735.52)	63.79 (6.42, 735.52)	51.50 (6.42, 735.52)	0.933
Sputum GSH (median, IQR, μmol/L)	240.45 (189.74, 392.60)	241.16 (189.74, 392.60)	234.69 (189.74, 422.57)	0.894
Sputum SOD (median, IQR,U/mL)	77.22 (16.86, 252.77)	79.80 (17.40, 241.18)	75.75 (16.56, 266.13)	0.835
Sputum CAT (median, IQR, U/mL)	1337.29 (246.73, 5012.41)	1566.75 (265.64, 5012.41)	737.17 (195.04, 5110.75)	0.155
Sputum TAC (median, IQR, U/mL)	74.75 (36.99, 185.28)	71.57 (38.37, 185.28)	86.21 (35.40, 185.28)	0.698
Sputum MDA (median, IQR,μmol/L)	6.19 (3.03, 17.54)	6.19 (2.77, 17.54)	7.22 (3.48, 17.54)	0.568
Sputum H2O2 (median, IQR, μmol/L)	197.24 (52.71, 286.27)	157.30 (54.73, 286.27)	216.48 (41.07, 286.27)	0.663
Blood GSH (median, IQR, μmol/L)	14.36 (11.60, 26.35)	20.47 (11.60, 29.58)	12.06 (11.60, 21.22)	0.003
Blood SOD (median, IQR, U/mL)	3.28 (1.56, 8.60)	3.40 (1.56, 8.07)	2.80 (1.56, 9.21)	0.79
Blood CAT (median, IQR, U/mL)	68.76 (21.36, 223.08)	75.32 (42.29, 240.23)	51.62 (18.33, 221.31)	0.004
Blood TAC (median, IQR, U/mL)	17.64 (15.98, 34.33)	17.64 (15.98, 35.27)	16.92 (15.98, 31.70)	0.185
Blood MDA (median, IQR, μmol/L)	2.52 (0.93, 4.70)	2.46 (0.92, 4.70)	2.66 (0.94, 4.70)	0.94
Blood H_2O_2(median, IQR, μmol/L)	3.79 (2.02, 6.12)	3.64 (2.22, 6.12)	4.85 (1.73, 6.37)	0.727
Blood Superoxide Anion (median, IQR, μmol/L)	76.56 (14.64, 597.08)	58.37 (14.64, 452.94)	116.63 (13.04, 597.08)	0.393

Definition of abbreviations: NO_2 = nitrite; NO_3 = nitrate; GSH= glutathione; SOD= superoxide dismutase; CAT= catalase; TAC= total antioxidant capacity; MDA= malondialdehyde; H2O2= hydrogen peroxide

Data presented as media (IQR) where applicable.
